# Stressor-Specific Microbiota Intervention

**DOI:** 10.3389/fnut.2022.870665

**Published:** 2022-04-18

**Authors:** Jie-Yu Chuang

**Affiliations:** ^1^Department of Psychiatry, Cardinal Tien Hospital, New Taipei City, Taiwan; ^2^School of Medicine, College of Medicine, Fu Jen Catholic University, New Taipei City, Taiwan

**Keywords:** stressor, microbiota, microbiome, brain inflammation, depression, PTSD, microglia

## Abstract

To date, mental disorders are diagnosed and treated by the subjective judgment of psychiatrists based on diagnostic criteria and treatment guidelines, respectively. Mental disorders are heterogeneous illnesses with a substantial treatment-refractory rate. Thus, there is a great need for novel treatment approaches. This article proposes a treatment approach centered on the concept of the gut–brain axis. There is mounting evidence indicating an association between stressors, microbiota, microglia, and mental disorders. Stressors might facilitate dysbiosis, inflammation, and the occurrence of mental disorders. This novel treatment approach is based on the idea that stressor types instead of the heterogeneous psychiatric diagnosis might be closer to the neurobiological underpinnings of mental disorders. First of all, patients with treatment-resistant mental disorders will be asked to describe their major stressors. Then, clinicians will calculate the total threat score and the total deprivation score. Subsequently, treatment tailored to the major stressor type will be administered to restore a healthy gut microbiome. Presumably, treatment will be aimed at increasing microbiota diversity in those who mainly have deprivation stressors and boosting *Actinobacteria* in those who have mainly threat stressors. Large-scale clinical trials are warranted to test this hypothetical approach.

## Introduction

Accumulating data indicate a complex link among stressors, aberrant gut microbiota, microglia, inflammation, and neuropsychiatric disorders. First, the bidirectional crosstalk between the gastrointestinal tract and the brain, the gut–brain axis, has been extensively researched in recent years. Communication between the gut and the brain occurs through the nervous, neuroendocrine, and immune systems (1). Second, despite an undetermined mechanism, there is an evidence of a link among stressors, microbiota dysbiosis, and inflammation. Stress can activate the hypothalamic–pituitary–adrenal (HPA) axis and trigger cortisol release, which affects intestinal barrier integrity and alters the microbiota composition (2). Stress may result in a leaky gut that allows bacteria to seep into the circulation and induce inflammation (3). Immune cells can act as messengers that convey stress signals to the gut (3). This stress might deactivate the executive function in response to food cues and elicit a bias toward an unhealthy diet, thus affecting the gut microbiota (3). Reciprocally, the gut microbiota may modulate brain activity. Third, chronic inflammation and microbiota dysbiosis related to various stressors might be associated with neuropsychiatric disorders, such as depression (4). Possible mechanisms linking inflammation to neuropsychiatric disorders include cytokine-mediated stimulation of indoleamine 2,3-dioxygenase to retard serotonin production; cytokine-mediated oxidative stress and glial cell damage in the prefrontal cortex and amygdala; cytokine-mediated glutamate dysregulation and excitotoxicity, leading to reduced brain-derived neurotrophic factor (BDNF) production; inflammation-induced glucocorticoid resistance and decreased inhibitory feedback of corticotropin-releasing hormone (CRH); and cytokine-mediated intensification of the stress response (5). Finally, microglia and the gut microbiota might communicate *via* signal transduction through the vagus nerve (6) and circulation (7). The gut microbiota might alter the permeability of the intestinal barrier, permitting entry of pro-inflammatory cytokines, or gut-derived metabolites (e.g., acetylcholine, gamma-aminobutyric acid [GABA], serotonin, and short-chain fatty acids [SCFAs]) into the circulation, thus compromising the integrity of the blood–brain barrier and influencing microglia (6). Indeed, microglia are the first responders to neuroinflammation as they rapidly adapt their functions in response to the brain milieu (7). Neuroinflammation might trigger microglia to release pro-inflammatory cytokines, resulting in more neuronal damage (8). Moreover, it has been proposed that aberrant communication between the microbiota and the microglia might be related to neuropsychiatric symptoms, eventually leading to neurodegeneration (7).

Notably, inflammation is neither necessary nor sufficient to induce or sustain neuropsychiatric disorders, and only about one-third of patients with depression have higher inflammation than controls (4). However, excessive inflammation was significantly associated with treatment resistance in many psychiatric disorders, such as major depressive disorder (9), mood disorders (10), and schizophrenia (11). In this article, a novel approach to treatment-resistant mental disorders based on the bidirectional crosstalks between stressors, microbiota, and microglia is proposed. In the following text, the proposed approach will be elucidated after the introduction of each player (i.e., stressors, microbiota, and microglia).

## Stressors

In 1936, Hans Selye reported a non-specific bodily response to diverse nocuous agents that he named “general adaptation syndrome,” which is known as stress (12). Later, he named the factor triggering the stress response a “stressor” (12). Evidence suggests that the effects of a stressor on neurobiological systems are not related to the features of the stressor but to an individual's perception and interpretation of the stressor (13). For instance, the cortisol response to social speech stress is associated with perceived stress (14). Stress exposure increases the risk of developing a broad range of psychiatric disorders, including major depressive disorder and post-traumatic stress disorder (PTSD), which is closely related to stress (15).

Different types of stressors differentially affect brain responses, behaviors, and symptomology (16). Several stressor classifications have been proposed. Frank et al. suggested that acute stress induces a transient proliferation of microglia, whereas chronic stress causes apoptosis of microglia and consequent reduction in microglial cell numbers in rodents (17). This finding indicates that acute and chronic stress might have distinct impacts on the brain. Based on the HPA axis response after exposure to stressors in rodents, Sandi and Haller described two types of stressors, namely, stressors related to a decline in HPA axis activity (e.g., early deprivation, early subjugation, and peripubertal stress) and stressors related to normal HPA axis activity (e.g., post-weaning social isolation) (18). In humans, chronic and/or extreme stress can cause more significant consequences (13). Furthermore, stressors can be categorized as early or late in life, acute or chronic, and macro or micro (19). Wheaton and Montazer classified stressors as conditions of threat, challenges, demands, and structural constraints that call into question the operating integrity of the human beings (19).

Recently, considerable research has been conducted on early life stress because of its enormous impact on mental and physical health. The two predominant models of early life stress are the general or lumping model and the specific or splitting model (13). In the general model, stressors are lumped and treated as a broad category (13). In the specific model, different types of stressors are assumed to have distinct effects (13). For example, stressors can be classified as a lack of expected inputs (i.e., a deprivation-type stressor, such as neglect or food shortage) or a presence of a direct threat (i.e., a threat-type stressor, such as abuse or violence) (13). Nevertheless, there is no consensus on whether the general or specific model of early life stress is favored (13). Sheridan and McLaughlin further proposed the dimensional model of adversity and psychopathology (DMAP), stating that most adverse childhood experiences are complex exposures to co-occurring deprivation and threat stressors (20). For example, institutionalization might involve both neglect (deprivation) and abuse (threat) (20). Several studies indicate that deprivation and threat stressors differentially affect the human brain. For example, opposing influences of deprivation and threat on the structural integrity of the stria terminalis in young adults have been observed, with less generalized fractional anisotropy associated with greater threat and less socioeconomic deprivation (21). It has been speculated that threat stressors tend to be acute and diminish brain structural integrity *via* excitotoxic effects of glucocorticoids, whereas deprivation stressors tend to be chronic and strengthen brain structural integrity *via* coordinated activation (21). Furthermore, deprivation was found to be negatively associated with executive function in early childhood, whereas threat was not associated with executive function (22).

## Microbiota

Trillions of microbes reside in the human gut. In adults, the major phyla are Bacteroidetes and Firmicutes, and the minor phyla are Actinobacteria, Proteobacteria, and Verrucomicrobia (23). Their relative proportions and microbiota species vary markedly across individuals (23). Gut microbiota is essential for healthy living. For instance, germ-free mice (without microbiota) have greater blood–brain barrier permeability than control mice, partially due to reduced expression of tight-junction proteins (1). Among the Bacteroidetes, a higher ratio of *Prevotella* to *Bacteroides* correlates with higher microbiota diversity (23). Compared with healthy people, lower microbiota diversity has been reproducibly observed in patients with inflammatory bowel disease, atopic eczema, psoriatic arthritis, diabetes mellitus, arterial stiffness, and obesity (24). The link between reduced microbiota diversity and disease might indicate that a species-rich gut ecosystem is more robust against environmental hazards (24). Indeed, lower microbiota diversity is a marker of dysbiosis (imbalance of the microbiome) (24). In addition to lower diversity, dysbiosis can take many different forms, e.g., a reduction of anaerobes, an increase of facultative anaerobes (25), loss of keystone taxa, shifts in metabolic capacity, or blooms of pathogens (26). In spite of the fact that there is no consensus on what defines a healthy gut microbiota, current evidence suggests that a healthy gut consists of a diverse and well-balanced microbiota (2).

To date, there was minimal cohesion in human microbiota studies with few reliable replicated findings, possibly due to small sample sizes, confounding factors, and unstandardized methodologies (27). Results from two recent systematic reviews across psychiatric disorders indicate that microbiota diversity failed to show a significant difference between patients with mental disorders and healthy controls (27, 28). Furthermore, it has been suggested that less evidence of mental disorder diagnosis specificity was found in microbiota research (28). Instead, a transdiagnostic pattern of microbiota signature was found (27, 28). Namely, at the genus level, a lower abundance of the SCFAs producer *Faecalibacterium* was found in patients with mental disorders compared to healthy individuals (27, 28).

Although there is a heritable component of the gut microbiota, environmental factors are the major determinants of its composition (24). Examining genotype and gut microbiome data from 1,046 healthy Israeli adults, Rothschild et al. found that host single nucleotide polymorphisms could not be used to infer a statistically significant fraction of variability. They concluded that 20.03% of inter-person microbiome variability is associated with factors related to diet, drug use, and anthropometric measurements (29). In fact, significant changes in the gut microbiota occurred within days of dietary alteration (24). Furthermore, it has been found that temporal within-individual microbiome variability, attributed to factors, such as diet, medication, and stool moisture, is substantially larger than inter-person microbiome variability (30). Consequently, future microbiome studies should adopt a repeated measurement design (30).

Although human studies are sparse, they suggest that SCFAs, mainly produced by the fermentation of dietary fiber by gut bacteria, are key mediators of the gut–brain axis (31). SCFAs have been implicated in various neuropsychiatric disorders, such as Parkinson's disease, Alzheimer's disease, autism, depression, anxiety, schizophrenia, and obesity (31). The most abundant SCFAs are acetate, propionate, and butyrate (31). These molecules perform a number of functions, including serving as energy sources for colonocytes and hepatocytes; maintaining intestinal barrier integrity to prevent systemic inflammation; increasing mucin secretion; modulating gut activity; inhibiting histone deacetylases to promote transcription; regulating the activation of neutrophils, dendritic cells, macrophages, monocytes, microglia, and T cells; stimulating the release of glucagon-like peptide 1 and peptide YY; activating vagal afferent nerve fibers; increasing BDNF production to promote neurogenesis; and stimulating serotonin synthesis (31). In rodents, SCFAs have demonstrated their ability to promote the maturation of microglia and reduce microglial activation (32). The levels of SCFAs are decreased in a naturally occurring macaque model of depression (30, 33). Furthermore, the antidepressant-like effect of SCFAs has been shown in mouse models (32). Nevertheless, human studies on the modulatory role of SCFAs in inflammation remain scarce, and the results are inconsistent (31). Additional human studies are needed to determine the association between SCFAs, microglia, microbiota, and inflammation. Fortunately, SCFAs can be quantified by gas chromatography–mass spectrometry (GC-MS) in feces and ion chromatography in blood (34). Similar to SCFAs, serotonin, GABA, and cortisol serve as mediators of the gut–brain axis with their production modulated by microbiota (35).

Both flow cytometry and 16S ribosomal RNA (rRNA) polymerase chain reaction (PCR) have been used to quantify microbial abundances (36). However, 16S rRNA PCR detects intracellular and extracellular DNA, whereas flow cytometry only quantifies intact microbial cells (36).

## Microglia

Microglia, the macrophages present in the brain, are an emerging focus of immune research in mental disorders (37). Microglia are uniformly present in the brain and represent 5–10% of brain cells. They function in self-defense, control of brain cell numbers, and refinement of neural circuits (37). Microglia can release either pro-inflammatory cytokines, such as IL-6, IL-12, IL-1β, and TNF-α, or anti-inflammatory cytokines, such as IL-4, IL-10, and TGF-β (6). Based on studies in mouse models, microglia can suppress overactive neuronal activity and are essential for preventing excessive activation in the brain (38).

Microglia are immunosensors of the stress response (17), and microglial activation in psychiatric patients is a potential marker of severity and is more likely to be associated with the effects of stressors rather than mental disorders (37). Continual input from a diverse gut microbiota is required for microglial maturation (6). In particular, microbiota-derived SCFAs play a pivotal role in the regulation of microglial maturation (39). Traditionally, *in vivo* microglial activation is quantified by positron emission tomography (PET) imaging targeting the translocator protein 18 kDa (TSPO) (40). However, tracers targeting TSPO come with many limitations, such as non-specific binding, low signal-to-noise ratio, and low brain uptake (40). Therefore, an effort is being put into the search for new targets for PET imaging of microglial activation.

## Clinical Practice in Psychiatry

As the famous German psychiatrist, Emil Kraepelin stated in 1920 “Trying to understand another human being's emotional life is fraught with potential error. This is especially worrying as we have no objective yardstick for this confidence” (41). More than 100 years later, and despite advances in scientific understanding of mental disorders, clinical practice in psychiatry still largely relies upon a subjective decision from the clinicians (41).

To date, clinical diagnosis is still made based on categorical systems, such as the Diagnostic and Statistical Manual of Mental Disorders (DSM-V). Nevertheless, clinical diagnosis based on categorical systems exhibits high heterogeneity (42). Recently, Kelly et al. (43) proposed the integration of a microbiome signature as an additional component of the research domain criteria (RDoC), which promotes transdiagnostic dimensional constructs according to neurobiological measures. Furthermore, there is no validated biomarker in psychiatry, and treatment progress is monitored by clinical questionnaires. With mounting evidence indicating a role of inflammation in the etiology of mental disorders, Bullmore urges the search of a useful biomarker to trace brain inflammation (44) in patients with mental disorders. Peripheral blood inflammatory biomarkers, such as C-reactive protein (CRP), fail to reflect neuroinflammation precisely. However, severe headache can be caused by cerebrospinal fluid sampling (44). A specific and sensitive PET radiotracer for microglial activation as a proxy for brain inflammation might instead be anticipated (45).

## A Proposed Approach: Stressor-Specific Microbiota Intervention

Two recent reviews indicate less evidence of psychiatric disorder diagnosis specificity in microbiota composition (27, 28). However, decreased microbiota species richness in patients with anorexia nervosa and comparable microbiota species richness in patients with PTSD are the most consistently reported results in the literature (27). Intriguingly, according to the DMAP, patients with anorexia nervosa are likely to encounter mainly deprivation stressors, whereas patients with PTSD tend to encounter mainly threat stressors. These findings suggest that we should consider stressor types instead of psychiatric diagnosis in microbiota-gut-brain research. Borrowing the concept from the DMAP model (13), this study proposes a new approach for the treatment of mental disorders based on microbiota-gut-brain research. In short, patients with treatment-refractory mental disorders will be asked to describe their current major stressors. Clinicians will then calculate the total deprivation score and total threat score using methods described by Machlin et al. (46). Major stressor type will be determined by comparing between these two scores. Subsequently, to restore a healthy microbiome, treatment will be administered according to their major stressor type.

Several microglia-related studies support the proposed approach. In a rodent study, permanent social isolation (a deprivation stressor) and repeated injection (a threat stressor) exerted divergent effects on microglial cell density (16), indicating that microglia may be differentially affected by deprivation and threat stressors. A retrospective study showed that patients with PTSD are more likely to have been exposed to childhood sexual trauma (a threat stressor) than patients with major depressive disorder (47), indicating that unlike patients with depression, patients with PTSD are more likely to encounter threat stressors than deprivation stressors. In contrast to elevated microglial activation in major depressive disorder, reduced microglial activation was observed in PTSD, suggesting deficient neuroimmune neuroprotective function (48).

Microbiota may also be divergently influenced by deprivation and threat stressors. Deprivation stressors may be associated with reduced microbiota diversity. In a US study (Wisconsin Longitudinal Study), individuals who lived alone showed reduced microbiota alpha diversity (a measure of diversity within a sample) compared to married individuals (49). Another US study showed reduced alpha diversity in participants who are lonely (50). Loneliness was also associated with elevated levels of pro-inflammatory biomarkers (50). An analysis of fecal samples from 655 participants (77% from the USA) indicated that a small social network size was significantly associated with a lower alpha diversity index (51). Social isolation, extensive hygiene, and travel barriers during the COVID-19 pandemic may also reduce microbiota diversity (52). In contrast, threat stressors might be related to comparable microbiota diversity but significant changes in microbiota composition. A South African study showed similar microbiota alpha and beta diversities (differences between samples) but a decreased abundance of Actinobacteria, Lentisphaerae, and Verrucomicrobia in patients with PTSD when compared with trauma-exposed controls (53). In another study, a similar alpha microbiota diversity index with decreased abundances of Firmicutes and Actinobacteria was found in refugees (presumably under many threat stressors) compared with controls (54). Despite the paucity of studies, the significant findings converge on the phylum Actinobacteria, which constitute about 8% of the human microbiota, with *Bifidobacterium* as the dominant genus. *Bifidobacterium* species are major SCFAs producers and immune modulators (through induction of regulatory T cells) (55) that support the growth of other bacteria species (56). *Bifidobacterium* produces a high concentration of acetate which protects the host from enteropathogenic infections (55). Moreover, *Bifidobacterium* produces lactate, which can be metabolized by other bacteria to produce butyrate, the main energy source for colonocytes (55). Intriguingly, depletion of *Bifidobacterium* was found in patients with COVID-19, a severe threat to the entire population (57).

Based on these findings, it is hypothesized that threat stressors are more likely to be related to decreased abundance of *Actinobacteria*, whereas deprivation stressors tend to be associated with decreased microbiota diversity. Consequently, treatment will be aimed at increasing microbiota diversity in those who mainly have deprivation stressors and boosting *Actinobacteria* in those who have mainly threat stressors. However, large-scale clinical trials are needed to test this hypothesis. Additional validation of the proposed approach comes from genetic studies. Despite the complex big picture, different stressors seem to have dissimilar epigenetic effects (58). In a mouse model, social defeat stress (a threat stressor) induced the differential expression of a much higher number of genes compared to restraint stress (a deprivation stressor) (59). A systematic review of human studies also indicated divergent epigenetic changes related to these two types of stressors: methylation of spindle and kinetochore associated complex 2 (*SKA2*) was significantly associated with PTSD but not with depressive symptoms (58), and methylation of the glucocorticoid receptor gene *NRC31* was significantly associated with childhood physical neglect (a deprivation stressor) but not with physical abuse (a threat stressor) (60). Microbiota may modulate host transcription, alternative splicing, chromatin remodeling, DNA methylation, and histone acetylation (61). Nonetheless, the associations among stressors, microbiota, and genetic modifications remain elusive.

## Discussion

In summary, instead of using the traditional treatment approaches for heterogeneous mental disorders, this study proposes a novel approach that involves addressing the impact of core stressors on the microbiota, microglia, and inflammation, especially in patients with treatment-resistant mental disorders. Stressor types instead of the heterogeneous psychiatric diagnosis might be closer to the neurobiological underpinnings of mental disorders. Consequently, as compared to traditional treatment, this novel approach is anticipated to result in better treatment outcomes. Microbiome-based treatment will be tailored to the major stressor type in this new approach. Presumably, treatment will be aimed at increasing microbiota diversity in those who mainly have deprivation stressors and boosting *Actinobacteria* in those who have mainly threat stressors. Total threat score, total deprivation score, microglial activation, gut microbiota diversity, and gut microbiota composition can all be quantified. Moreover, to delve into the interaction between microbiota and microglia, the levels of SCFAs can also be quantified. Indeed, psychiatry of the future should be more objective and less subjective. Of note, the methods described by Machlin et al. (46) to calculate the total deprivation score and total threat score are initially designed for early life stress. Therefore, some changes might be needed for later life stress.

Several treatments can manage the impact of stress on the body. Minocycline has been shown to reduce microglial activation following stress exposure (37). Similarly, the beta-adrenergic blocker propranolol has been shown to reduce microglial activity and brain inflammation (62). Restoring healthy microbiota might alleviate the impact of stress. There are many microbiome-based therapeutics, e.g., fecal microbiota transplantation, diet and prebiotic supplementation, symbiotic microbial consortia transfer, engineered symbiotic bacteria transfer, and microbiota-derived metabolite supplementation (56). Prebiotics (non-digestible fiber) and natural probiotics promote gut microbial diversity (51). In particular, *Bifidobacterium* (phylum Actinobacteria) are widely used probiotics with many health benefits (55). Postbiotics are defined as inactivated microbiota with or without metabolites or cell components (63). Rifaximin, an intestinally acting antibiotic with low systemic absorption and minimal risk for provoking antibiotic resistance (64), exerts anti-inflammatory effects and enriches the beneficial microbiota (5). An increase in *Bifidobacterium* and *Lactobacillus* has been reported after rifaximin treatment (65). Moreover, a reduction of stressful perception of social exclusion was found after rifaximin ingestion in healthy adults (64). A Mediterranean diet, which is characterized by high intake of fruits, vegetables, and wholegrains and moderate intake of fish, poultry, and red wine, increased the levels of microbiota-derived SCFAs (7). Microbes have simpler genomes than humans; therefore, manipulation of their microbiome through gene-editing techniques, such as Clusters of Regularly Interspaced Short Palindromic Repeats (CRISPR), is a potential way to control stress responses (66). However, clinical studies are required to demonstrate the safety of such gene modifications.

Psychobiotics are defined as probiotics ingested that confer mental health benefits to the host through interaction with commensal gut microbiota (67). Despite a wide variety of research results, there is a lack of consensus in general (67). For example, animal studies related to psychobiotics are promising, yet human clinical trial results are slightly disappointing (63). Two clinical trials showed significant improvements in depressed symptoms at week 8 after probiotic supplementation with *Lactobacillus helveticus* and *Bifidobacterium longum* in patients with major depressive disorder (61, 68). However, probiotic supplementation with *Lactobacillus plantarum* failed to improve depressed symptoms at week 8 in another group of patients with major depressive disorder (69). Furthermore, prebiotic supplementation with galactooligosaccharide also failed to improve depressed symptoms at week 8 in depressed patients (70). Therefore, more clinical trials with extended follow-up durations are required. For the novel approach proposed in this article, treatment would be tailored to the major stressor type (i.e., deprivation or threat stressor). However, the interaction between stressor type and treatment choice has yet to be elucidated. Based on preliminary data, treatment-refractory individuals experiencing mainly deprivation stressors should be given treatments aimed at increasing microbiota diversity (i.e., oral ingestion of non-digestive fiber), whereas individuals who experience mainly threat stressors should receive treatments that increase Actinobacteria (e.g., oral ingestion of *Bifidobacteria* probiotics).

The field of microbiome research is young and exciting, with many unsolved mysteries. First, current gut–brain axis research is dominated by rodent studies. However, humans differ from rodents in many ways, underscoring the need for clinical studies (2). Most human studies have been observational or correlational, hindering the elucidation of the specific effects of different stressors (18). Moreover, human studies are complicated by each person's exposure to a unique combination of stressors, which is influenced by genetic and environmental factors (18). Second, in addition to the dominant bacteria, in future research, other microbes should also be considered. In most studies, only the predominant bacteria were analyzed. However, one study identified 1,952 uncultured candidate bacterial species in the gut (71). Furthermore, the gut microbiota comprises not just bacteria, but a wide range of archaea, eukaryotes, and viruses, which are awaiting exploration (71). Third, the role of sex differences in the gut–brain axis has yet to be revealed. Sex differences in immune responses to stress are well-established (17). Adult females exhibit more robust and prolonged physiological responses to stress (17). Moreover, female rats, but not male rats, have fewer microglia in the prefrontal cortex following restraint stress (17). Finally, whether or not microbiota interventions can alleviate neuropsychiatric symptoms in patients without aberrant inflammation, and whether or not it is possible to apply this novel, stressor-specific microbiota intervention to all patients with mental disorders requires further investigation.

## Data Availability Statement

The original contributions presented in the study are included in the article/supplementary material, further inquiries can be directed to the corresponding author.

## Author Contributions

J-YC developed the hypothesis and wrote the manuscript.

## Funding

J-YC was funded by Cardinal Tien Hospital.

## Conflict of Interest

The author declares that the research was conducted in the absence of any commercial or financial relationships that could be construed as a potential conflict of interest.

## Publisher's Note

All claims expressed in this article are solely those of the authors and do not necessarily represent those of their affiliated organizations, or those of the publisher, the editors and the reviewers. Any product that may be evaluated in this article, or claim that may be made by its manufacturer, is not guaranteed or endorsed by the publisher.
